# Supplementary material for: Changing travel patterns in China during the early stages of the COVID-19 pandemic
Source: Nat Commun. 2020 Oct 6;11:5012. doi: 10.1038/s41467-020-18783-0 (PMC7538915; doi:10.1038/s41467-020-18783-0)
Supplement: Supplementary file 1 — Supplementary Information [file 41467_2020_18783_MOESM1_ESM.pdf]

Supplementary Information

Changing travel patterns in China during the early stages of the COVID-19 pandemic

Gibbs et al

Supplementary Tables

Geographic and Demographic Characteristics of Prefectures by Cluster

Cluster	Number of Prefectures
A	22
B	34
C	33
D	36
Total	125

Supplementary Table 1. Number of prefectures per cluster.

Cluster	Distance from Wuhan (km)
A	259.85
B	505.31
C	707.27
D	829.48

Supplementary Table 2. Average distance to Wuhan from prefectures in each identified cluster. Distances were measured from the centroid location of each prefecture to the centroid location of Wuhan prefecture.

Cluster	Population Density (persons per km^2)
A	422
B	527
C	491
D	1233

Supplementary Table 3. Average population density of prefectures in each cluster. Population density was computed by dividing the population of each prefecture by the area of the prefecture, giving the number of persons per kilometer squared.

Supplementary Figure

K-Means Trajectory Clustering Analyses: Exploring the Number of Clusters

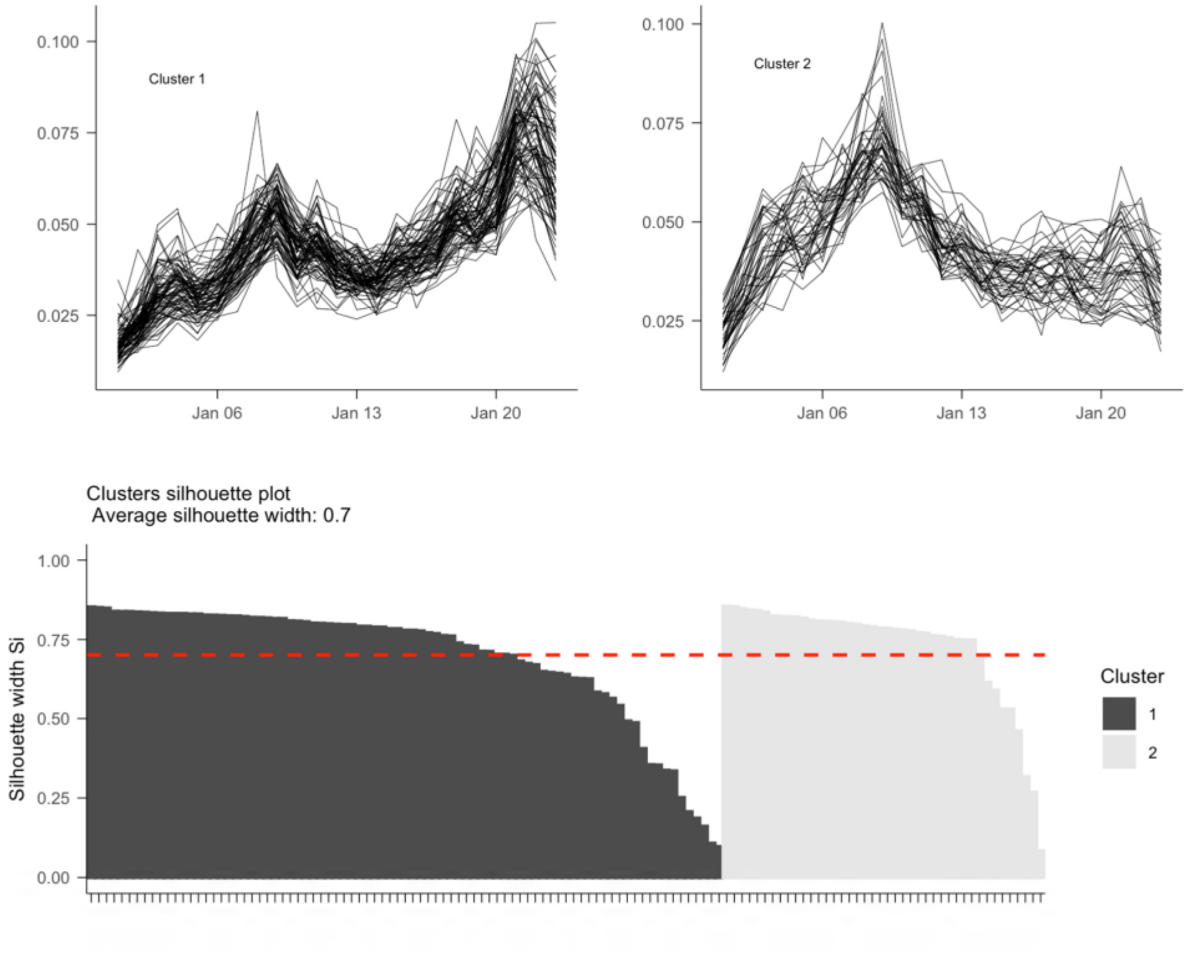

Supplementary Figure 1. Silhouette plot of outflow trajectories from Wuhan, clustered using K-means clustering with 2 clusters.

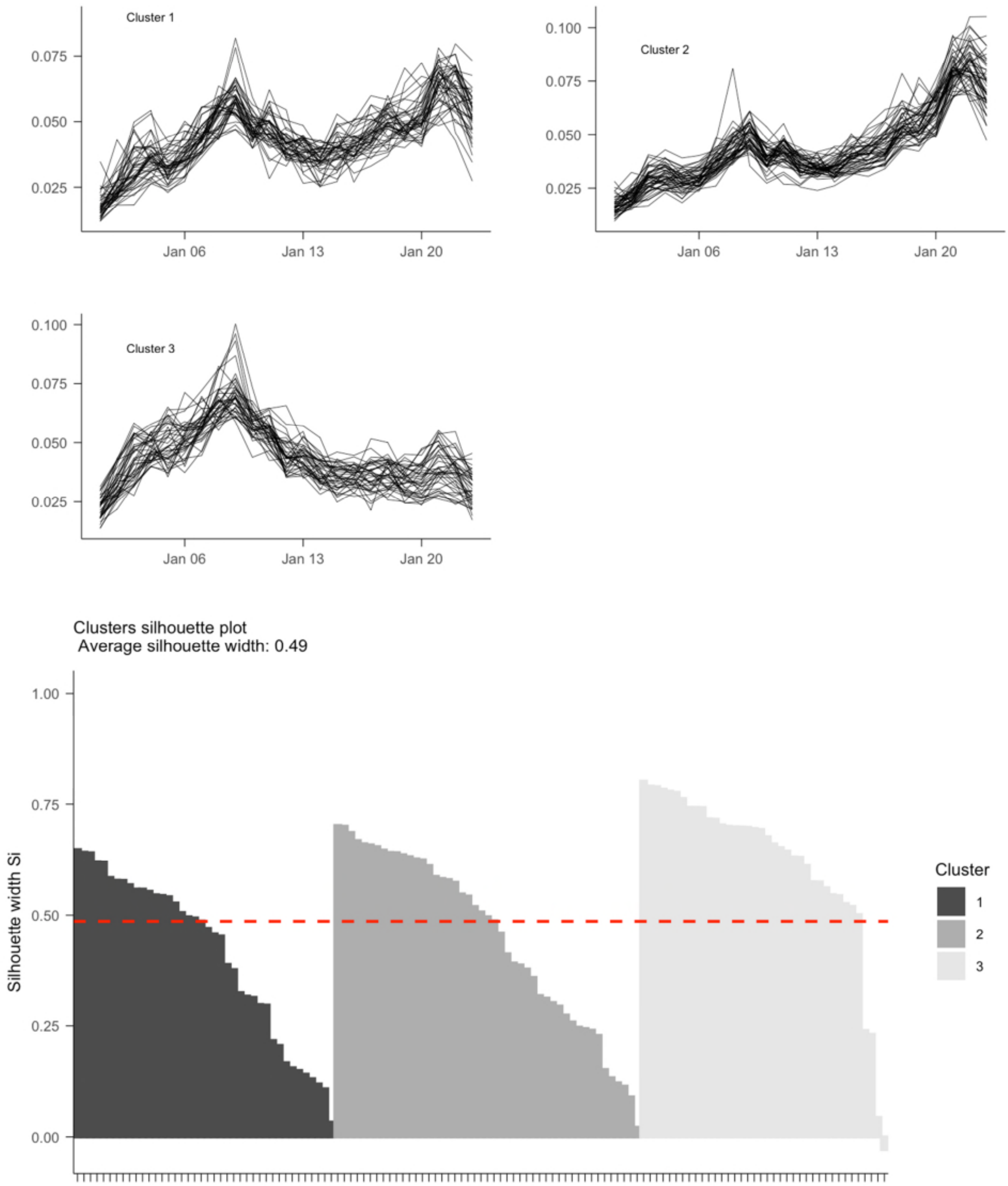

Supplementary Figure 2. Silhouette plot of outflow trajectories from Wuhan, clustered using K-means clustering with 3 clusters.

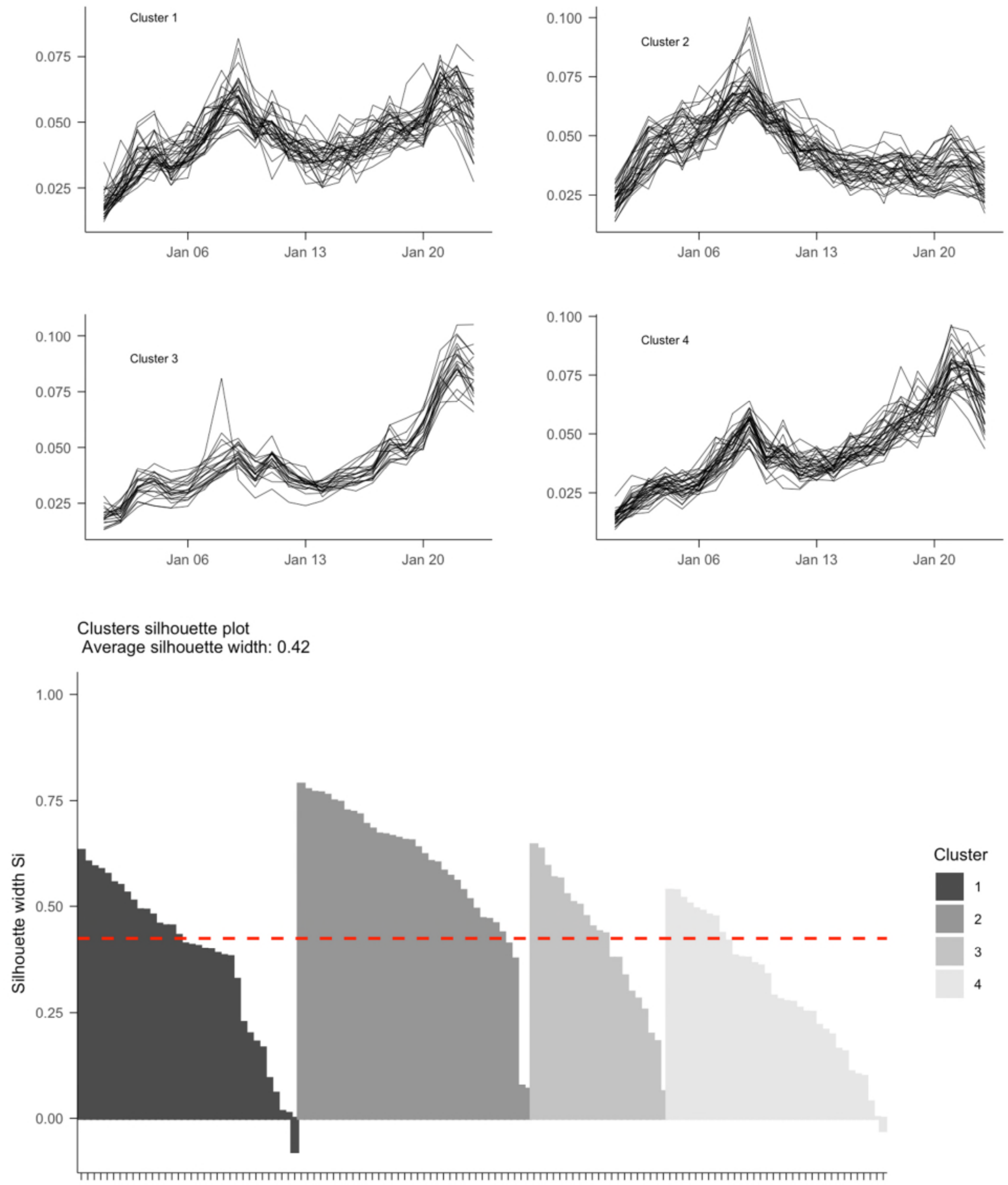

Supplementary Figure 3. Silhouette plot of outflow trajectories from Wuhan, clustered using K-means clustering with 4 clusters.

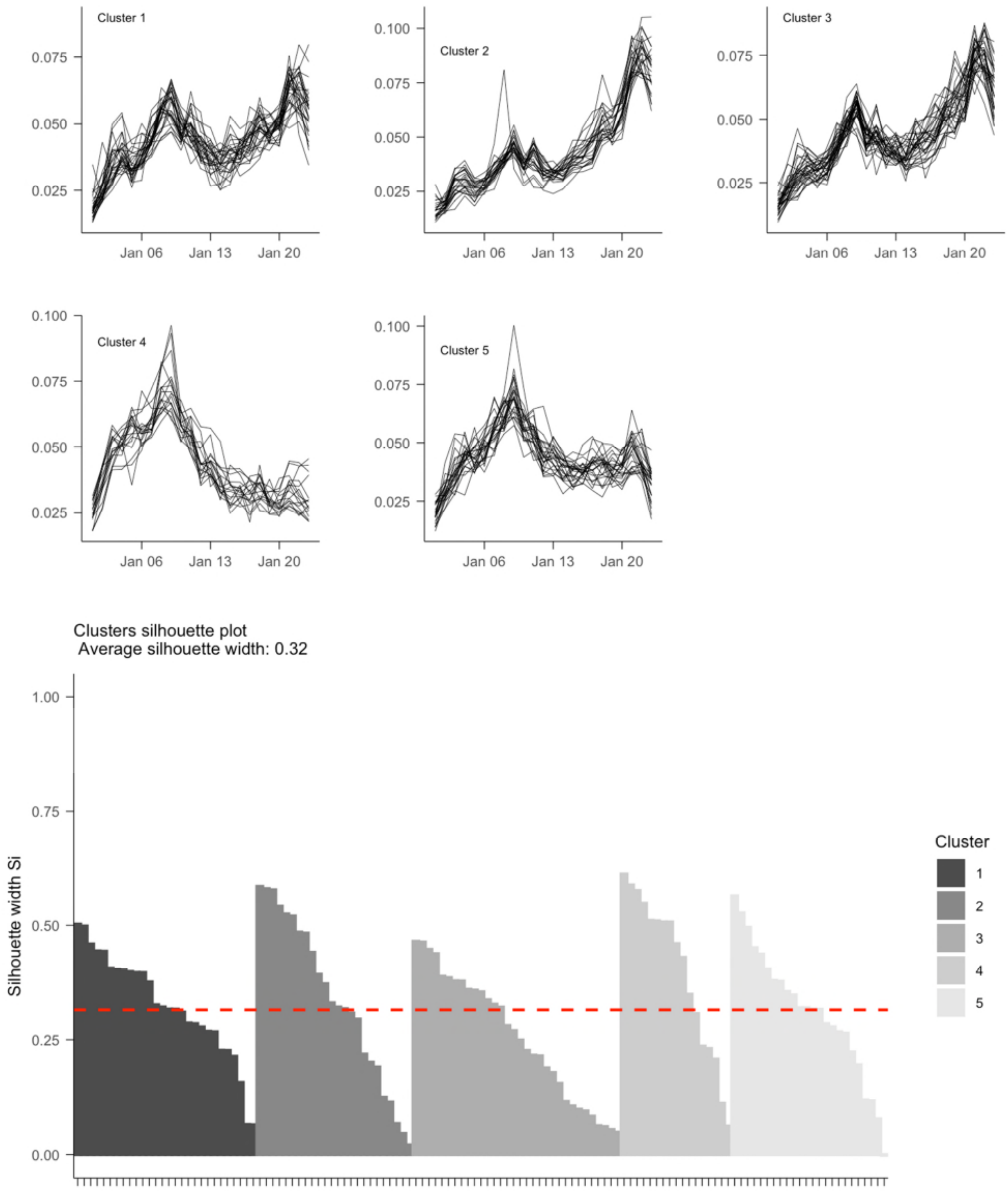

Supplementary Figure 4. Silhouette plot of outflow trajectories from Wuhan, clustered using K-means clustering with 5 clusters.

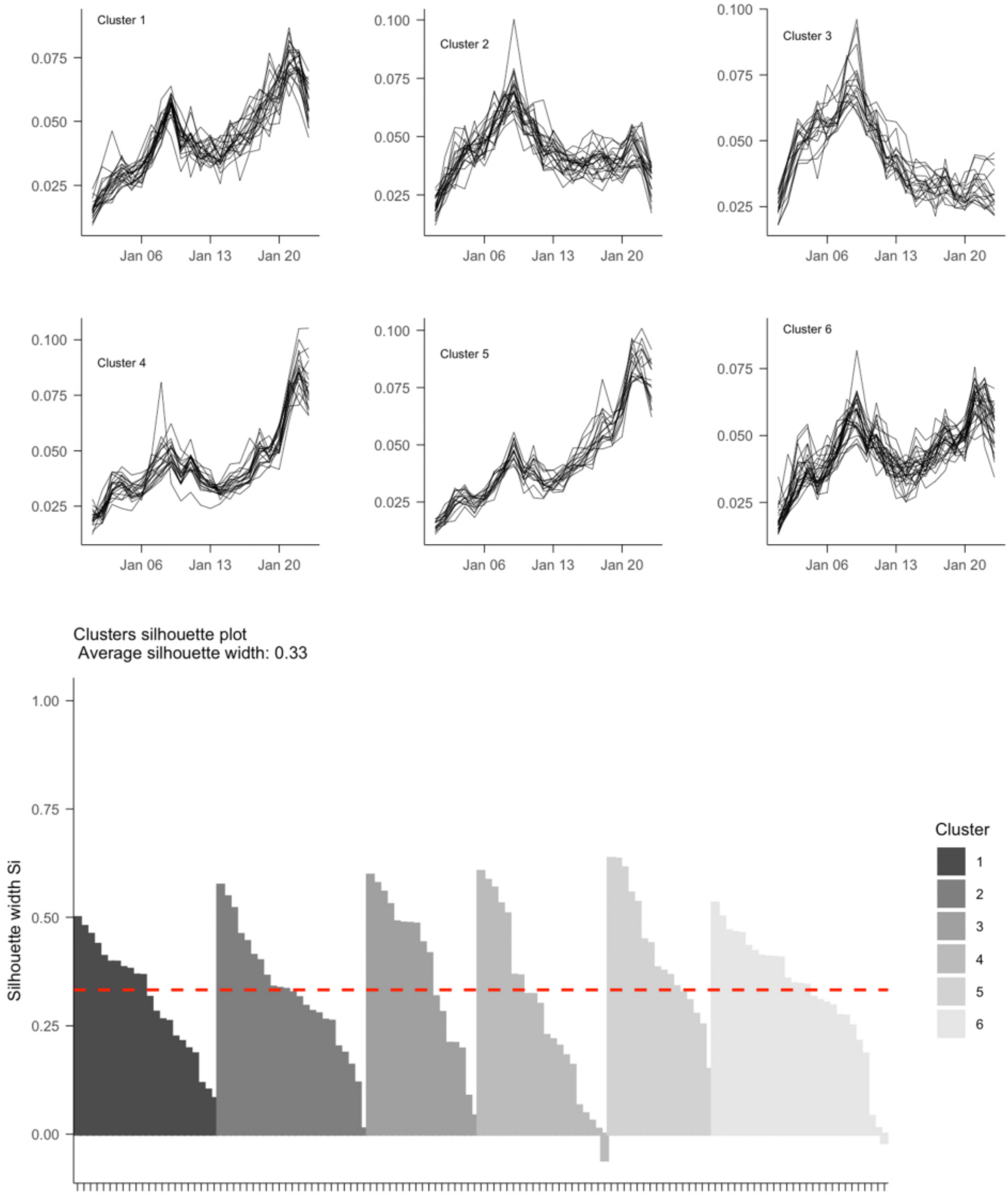

Supplementary Figure 5. Silhouette plot of outflow trajectories from Wuhan, clustered using K-means clustering with 6 clusters.

Understanding the Relationship between First Case Detection and Cluster Membership

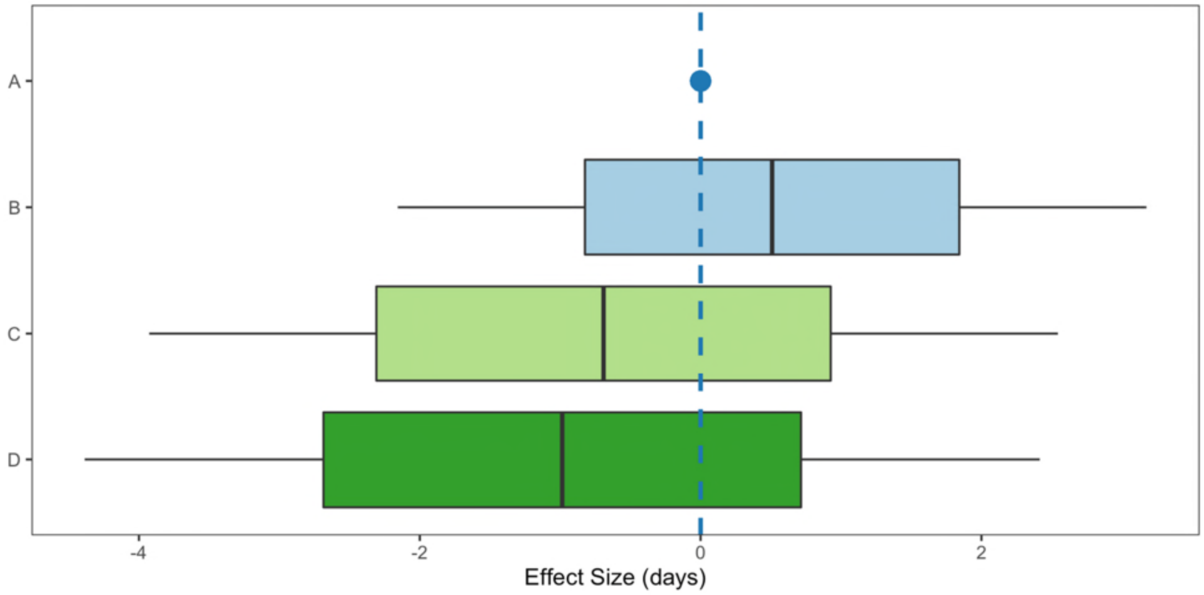

Supplementary Figure 6. Box plot of the effect size of the detection of first cases by cluster membership.

The effect size of detection of first case relative to cluster A for clusters B, C, and D. Boxplots show interquartile range and 95% confidence interval (B: -2.0167–2.9814, C: -3.5778–2.3481, D: -4.0883–1.9342). For linear model of effect size given cluster membership normalised by population: R^2 = 0.1769 (pictured). For linear model of effect size given cluster membership normalised by log of population: R^2 = 0.1654.. n = 22 (Cluster A), 34 (Cluster B) 33 (Cluster C), 36 (Cluster D). The data are displayed as Median, IQR, and whiskers +/- 1.5 times IQR.

Prefecture/ City specific K-mean Trajectory Clustering Elsewhere

The number of clusters was chosen by comparing silhouette plots of different numbers of clusters, using the same method as that used for trajectory clustering for outgoing travel from Wuhan.

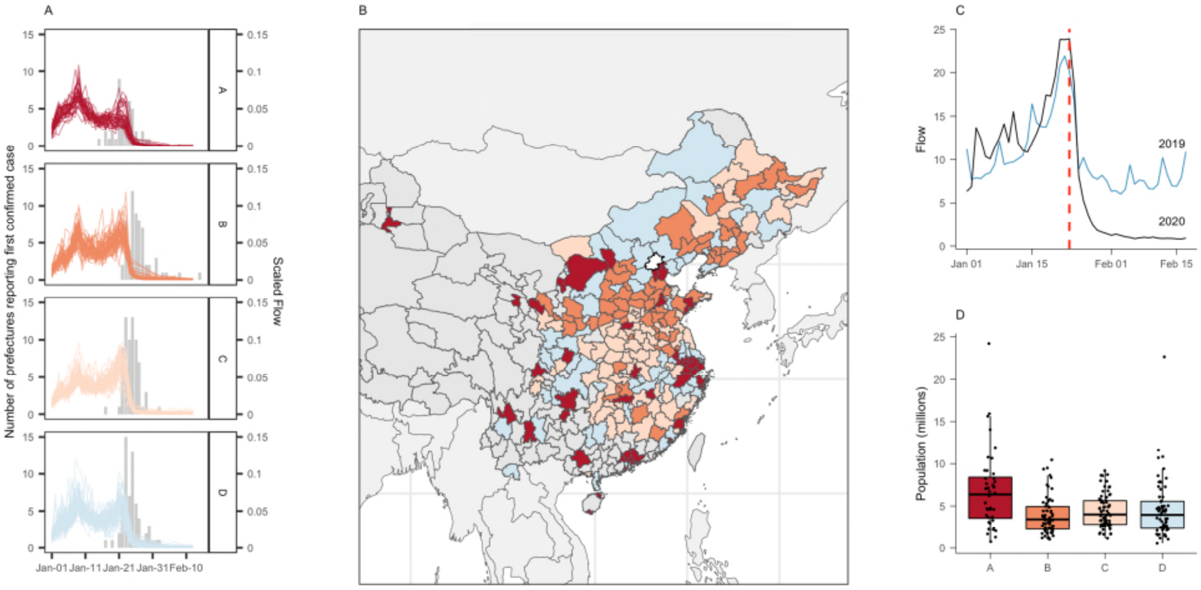

Supplementary Figure 7. Movement characteristics of travel originating in Beijing.

The identified patterns in outbound travel from Beijing: a) pairwise travel trends from Beijing to the most connected prefectures, stratified by cluster. Grey bars show the number of prefectures in that cluster with first confirmed case on each date; b), the spatial distribution of the prefectures in each of the 4 identified clusters; c), the volume of overall outbound travel on each day from Beijing in 2019 and 2020; d), resident population size of prefectures stratified by cluster. For clusters in panel d, n = 63 (Cluster A), 69 (Cluster B) 65 (Cluster C), 41 (Cluster D). The boxplot in panel d displays Median, IQR, and whiskers +/- 1.5 times IQR.

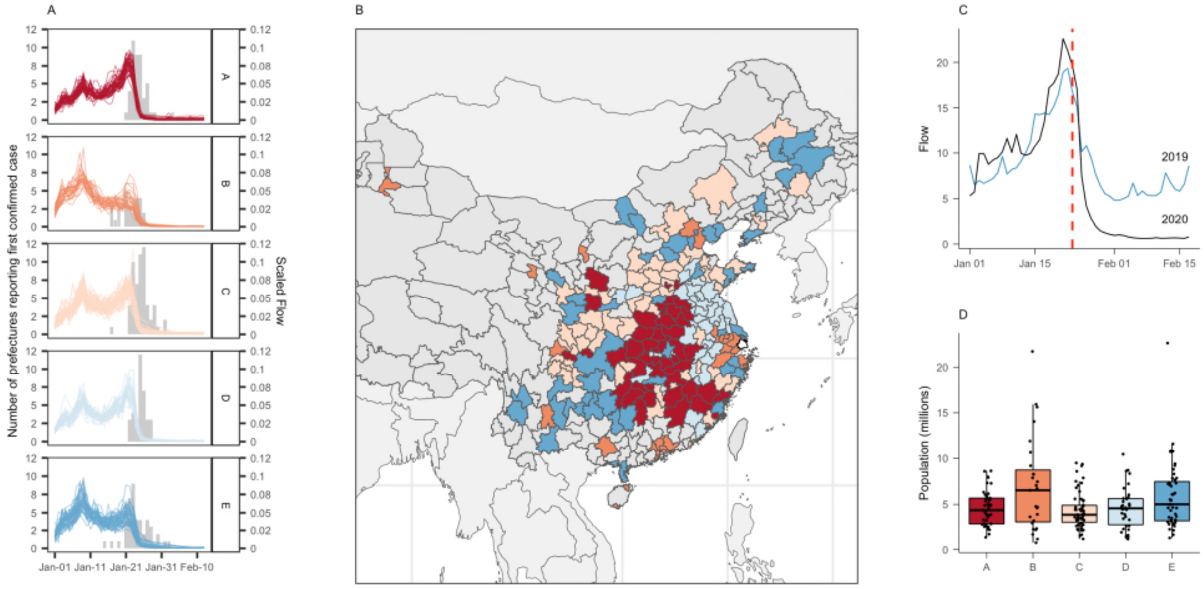

Supplementary Figure 8. Movement characteristics of travel originating in Shanghai.

The identified patterns in outbound travel from Shanghai: a) pairwise travel trends from Shanghai to the most connected prefectures, stratified by cluster. Grey bars show the number of prefectures in that cluster with first confirmed case on each date; b), the spatial distribution of the prefectures in each of the 5 identified clusters; c), the volume of overall outbound travel on each day from Beijing in 2019 and 2020; d), resident population size of prefectures stratified by cluster. For clusters in panel d, n = 45 (Cluster A), 27 (Cluster B) 58 (Cluster C), 34 (Cluster D), 49 (Cluster E). The boxplot in panel d displays Median, IQR, and whiskers +/- 1.5 times IQR.

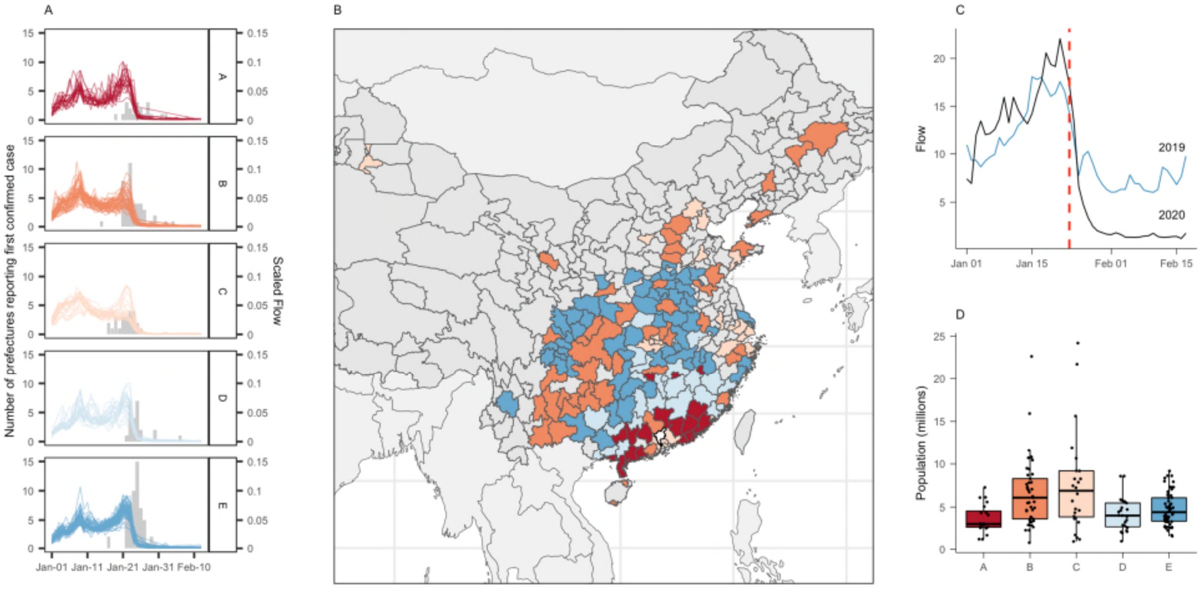

Supplementary Figure 9. Movement characteristics of travel originating in Guangzhou.

The identified patterns in outbound travel from Guangzhou: a) pairwise travel trends from Guangzhou to the most connected prefectures, stratified by cluster. Grey bars show the number of prefectures in that cluster with first confirmed case on each date; b), the spatial distribution of the prefectures in each of the 5 identified clusters; c), the volume of overall outbound travel on each day from Beijing in 2019 and 2020; d), resident population size of prefectures stratified by cluster. For clusters in panel d, n = 21 (Cluster A), 47 (Cluster B) 24 (Cluster C), 25 (Cluster D), 58 (Cluster E). The boxplot in panel d displays Median, IQR, and whiskers +/- 1.5 times IQR.

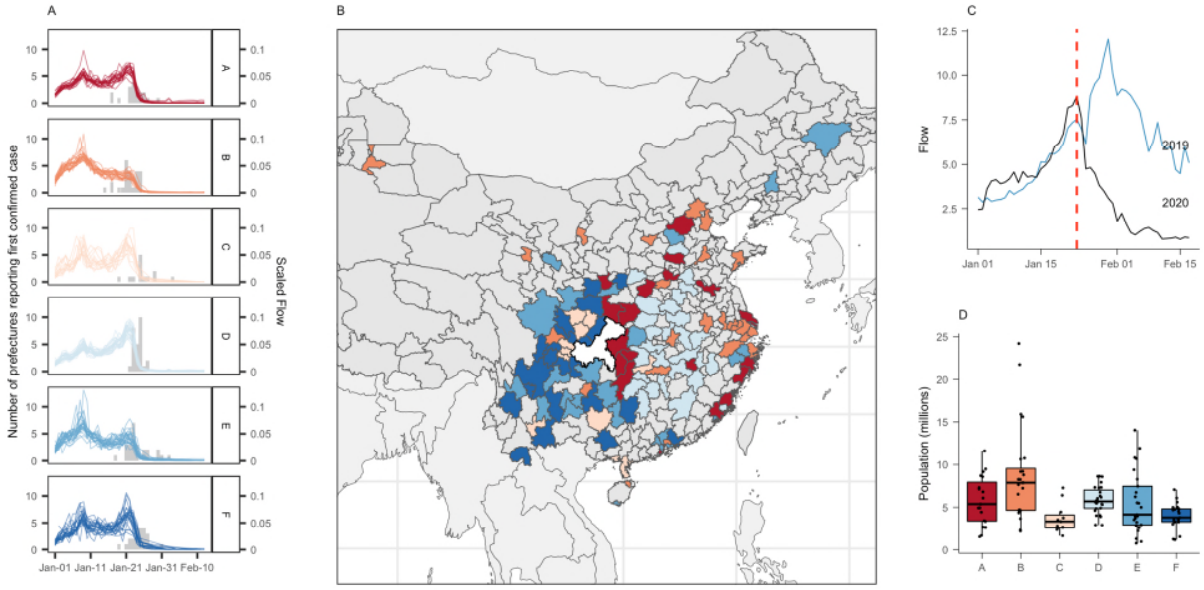

Supplementary Figure 10. Movement characteristics of travel originating in Chongqing.

The identified patterns in outbound travel from Chongqing: a) pairwise travel trends from Chongqing to the most connected prefectures, stratified by cluster. Grey bars show the number of prefectures in that cluster with first confirmed case on each date; b), the spatial distribution of the prefectures in each of the 6 identified clusters; c), the volume of overall outbound travel on each day from Beijing in 2019 and 2020; d), resident population size of prefectures stratified by cluster. For clusters in panel d, n = 20 (Cluster A), 24 (Cluster B) 13 (Cluster C), 27 (Cluster D), 26 (Cluster E), 23 (Cluster F). The boxplot in panel d displays Median, IQR, and whiskers +/- 1.5 times IQR.

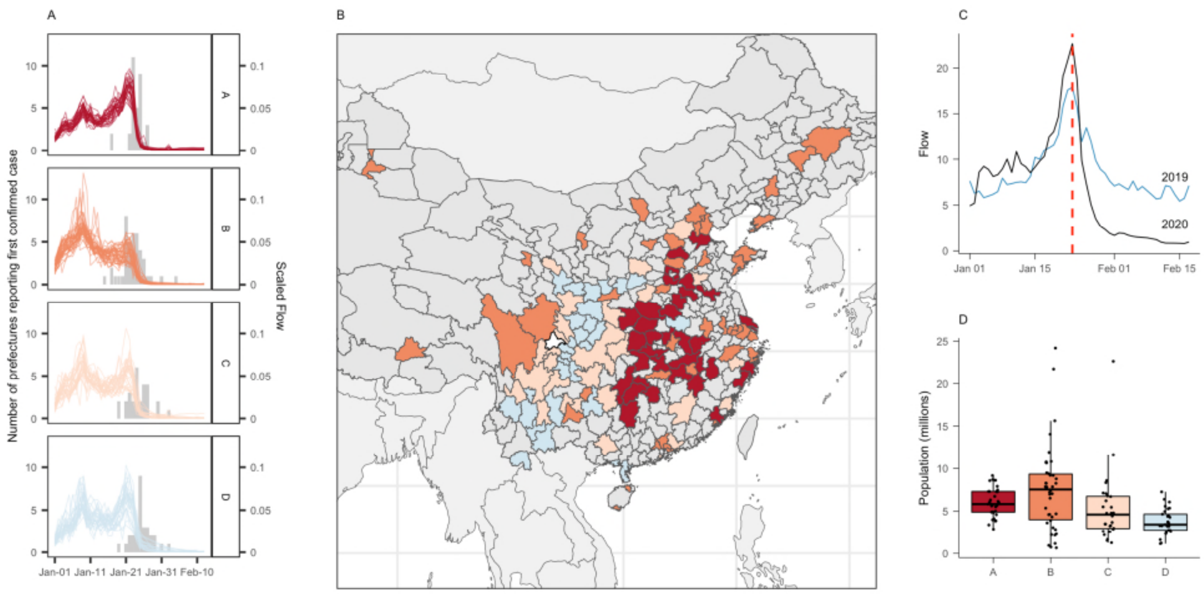

Supplementary Figure 11. Movement characteristics of travel originating in Chengdu.

The identified patterns in outbound travel from Chengdu: a) pairwise travel trends from Chengdu to the most connected prefectures, stratified by cluster. Grey bars show the number of prefectures in that cluster with first confirmed case on each date; b), the spatial distribution of the prefectures in each of the 4 identified clusters; c), the volume of overall outbound travel on each day from Beijing in 2019 and 2020; d), resident population size of prefectures stratified by cluster. For clusters in panel d, n = 33 (Cluster A), 42 (Cluster B) 30 (Cluster C), 30 (Cluster D). The boxplot in panel d displays Median, IQR, and whiskers +/- 1.5 times IQR.

Detecting outbound travel surges preceding LNY

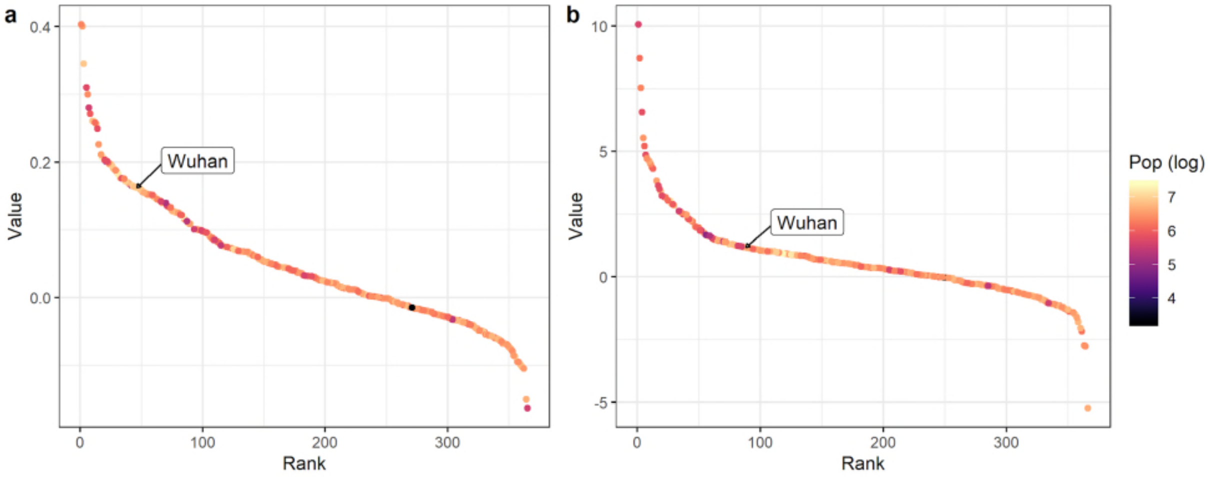

Supplementary Figure 12. The change in outbound travel surges between 2020 and 2019.

(a) shows the proportion-based variability metric eq.3 and (b) the anomaly-based variability metric eq. 4. Color gradient reflects local population sizes (log10-scaled).

The volume of in- and outbound travel between population quartiles in mainland China

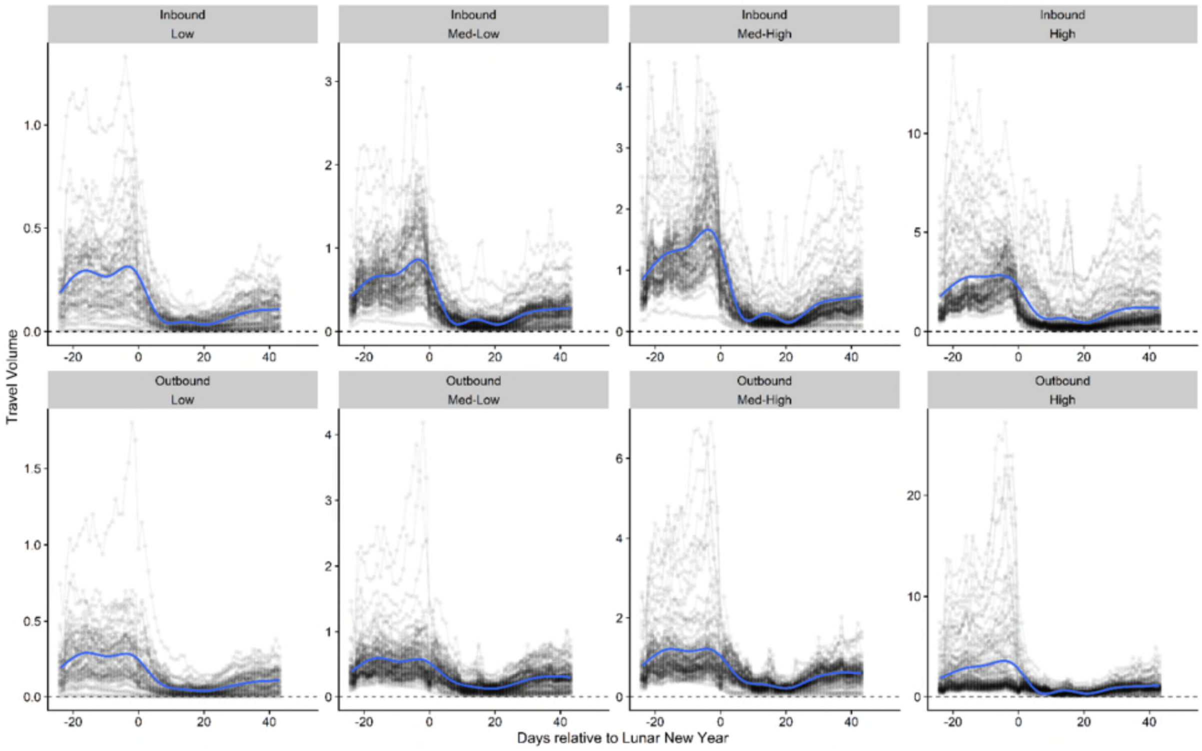

Supplementary Figure 13. In- and outbound travel volumes over time between population quartiles.

Travel volume is measured by the Baidu migration Index. Each line represents a prefecture. The blue line represents the average levels for a given direction, within a given population quartile. In- and outbound travel volumes show similar trends over time. Over the two to three weeks prior to the Lunar New Year (LNY), travel activities stablised at relatively high levels. Then, during the week after LNY, travel activity significantly dropped. As time goes on, travel activities gradually recover. By the end of this study, travel volumes have not been able to recover to their pre-LNY/ COVID-19 levels. Despite of similar trends, the specific compositions of these travel activities are distinctive based on population size and travel directions. More discussion can be found in the main text, centered around Figure 2.

Distance Kernel Plots for Large Cities in Mainland China

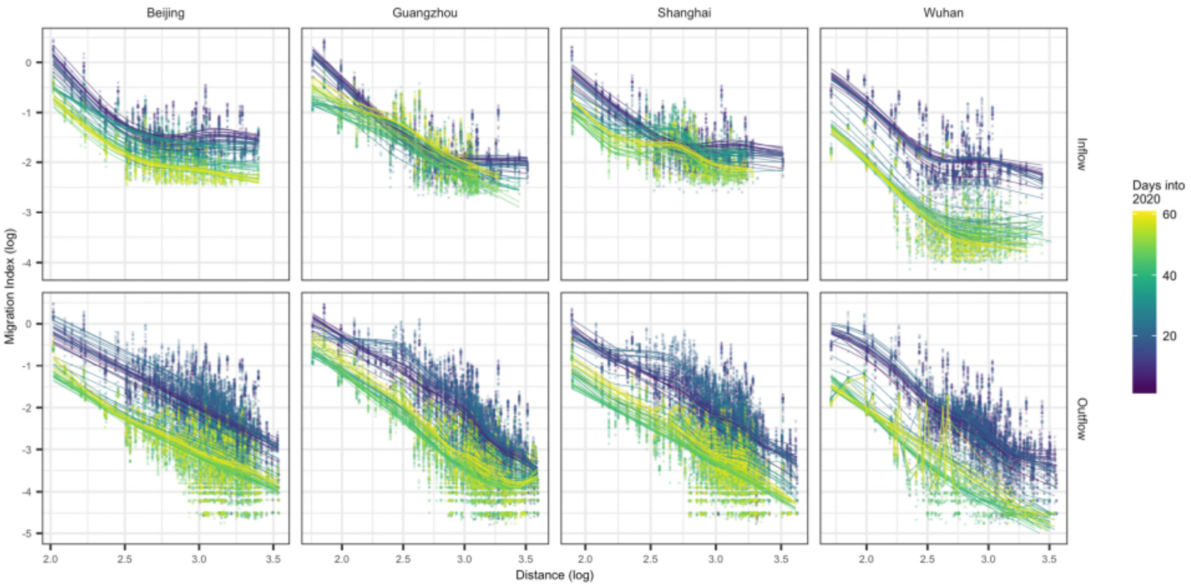

Supplementary Figure 14. Distance kernel plots for Beijing, Guangzhou, Shanghai and Wuhan.

The points show the frequency of journeys (y-axis) of at least the distance shown on the x-axis. The line is a smoothed spline. The colour shows the days since Jan 1 2020.

Weekly Pair-wise Mobility by Population Quartile

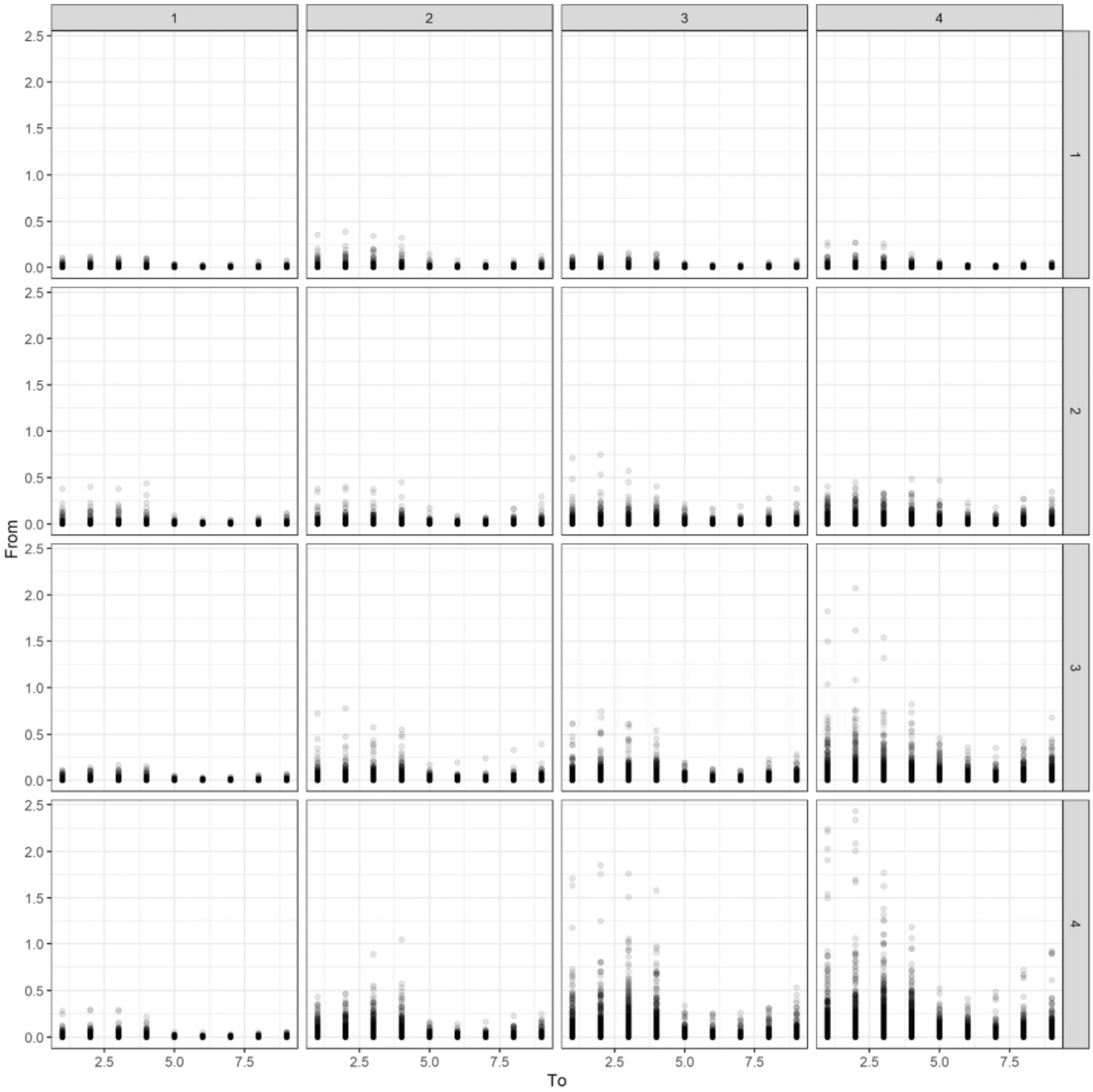

Supplementary Figure 15. Weekly pair-wise mobility by population quartiles. Index 1-4 are lowest-highest population size quartiles.

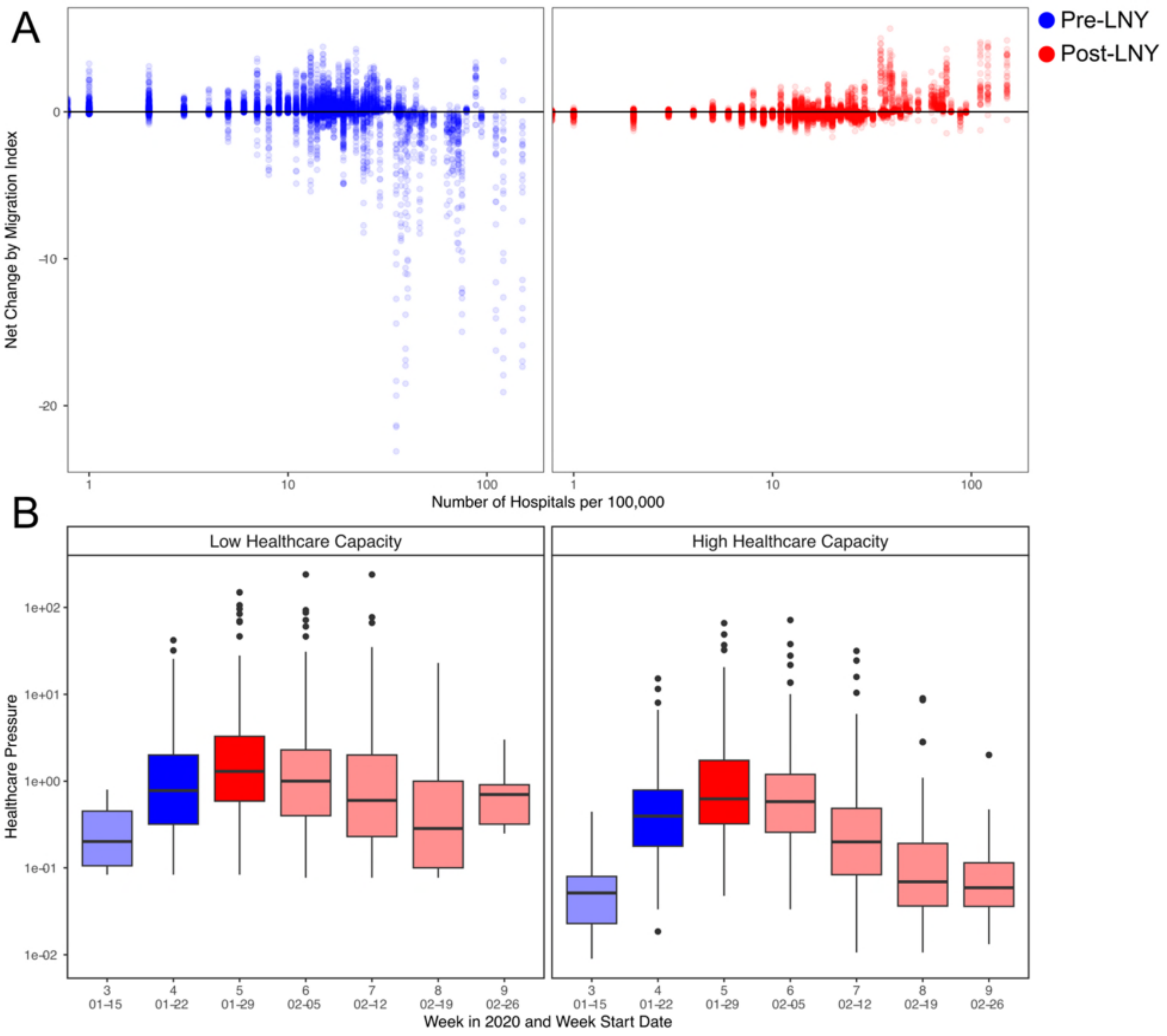

Supplementary Figure 16. Measuring healthcare capacity.

Assessing healthcare capacity related to COVID-19 using an alternate metric, the number of Grade II and Grade III hospitals in each prefecture, without adjusting for population size (n_{low} = 154, n_{high} = 156). A) The changes in traveller volume before (blue) and after (red) LNY. B) The changes in the healthcare pressure (log10 scale) related to COVID-19 each week in low and high healthcare capacity prefectures. Similar to figure 3 in the main text, the statistical significance (shown in this figure as shades, i.e., darker color indicates statistical significance, is based on a one-tail Mann Whitney U tests (n1 = 154, n2 = 156). The null hypothesis was that the healthcare pressures in settings with fewer hospitals are comparable to that in settings with more hospitals; the alternative hypothesis was that healthcare pressure in settings with fewer hospitals are higher than those in settings with more hospitals. This test was repeated for each week from week three to nine (i.e., starting on 15 Jan 2020). Results were verified using two-tailed Mann- Whitney U tests, as well as one-tailed Mann-Whitney U tests with the opposite null hypotheses. In this process, we found that in week 9, healthcare pressure in places with more hospitals is higher. Pre-LNY travels may

no longer drive COVID-19 related healthcare pressure later on. The boxplots in panel B display Median, IQR, and whiskers +/- 1.5 times IQR.

Community Modularity

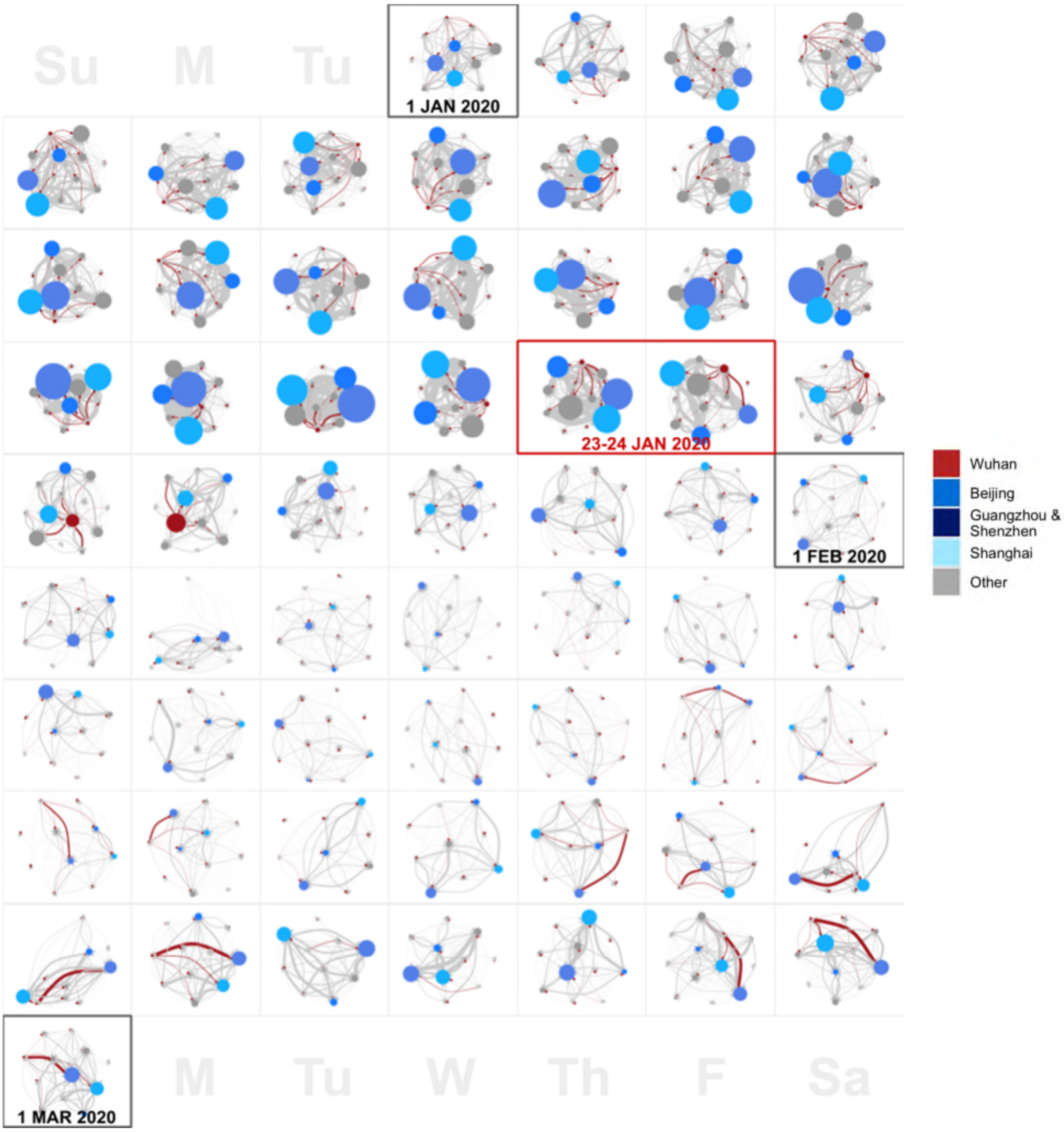

Supplementary Figure 17. Full Community Modularity Time Series.

Nodes combine all of the prefectures by community. Node size reflects intra-community travel, while edge size reflects extra-community travel. The red node includes Wuhan, and red edges represent the travel from that community. The blue nodes correspond to communities including major cities: Beijing, Shanghai, and Guangzhou &

Shenzhen (always appearing in the same community). The network plots appear on their corresponding calendar days.

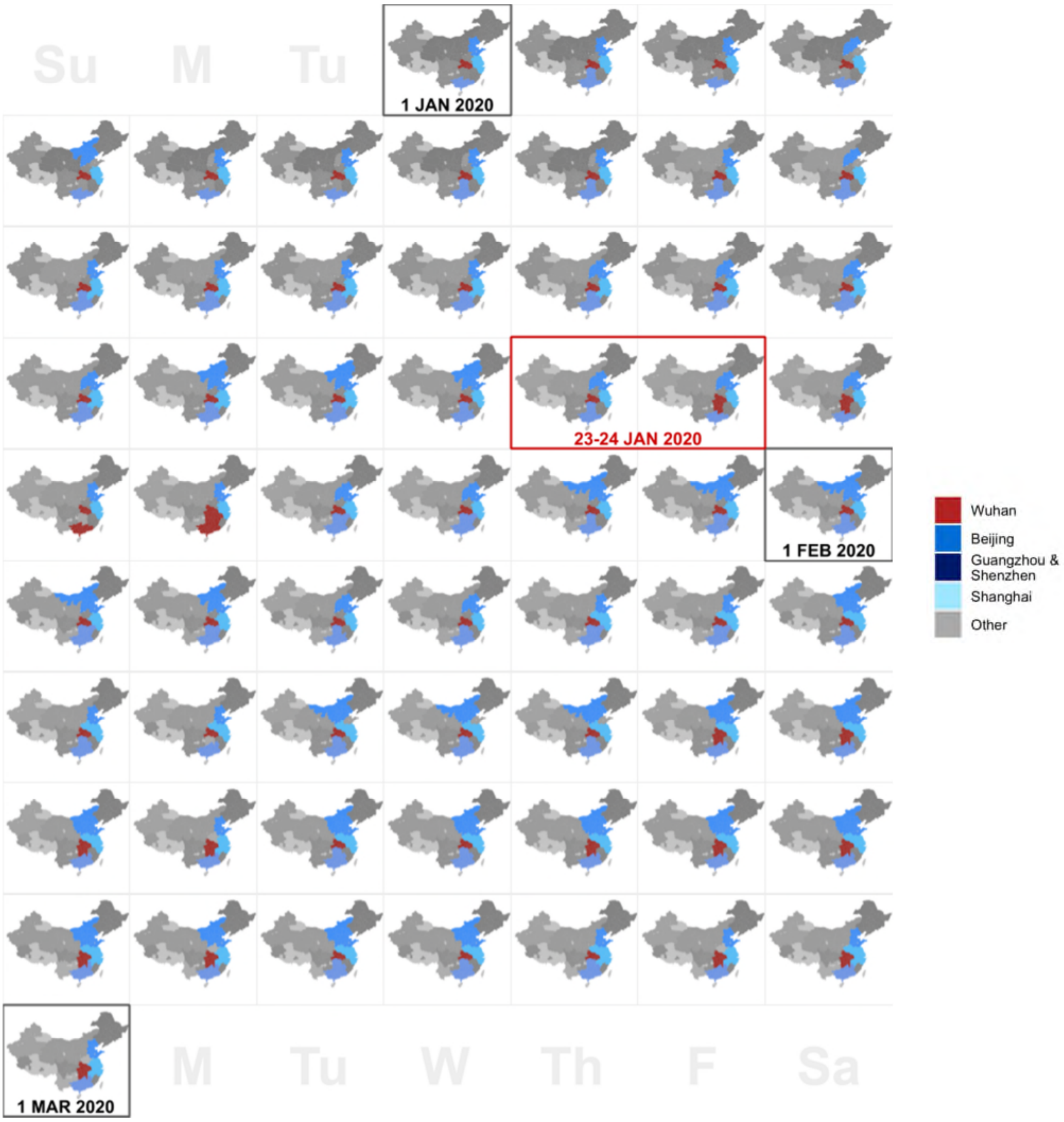

Supplementary Figure 18. Full Community Map Time Series.

These maps show the changing geographical regions associated with the identified communities. The coloring scheme is shared with Fig. 17, and again the small multiples appear on their corresponding calendar days. Prefectures coloured white are those where we have no mobility data, likely due to censoring due to low numbers of travellers.
