# Supplementary material for: Changing travel patterns in China during the early stages of the COVID-19 pandemic
Source: Nat Commun. 2020 Oct 6;11:5012. doi: 10.1038/s41467-020-18783-0 (PMC7538915; doi:10.1038/s41467-020-18783-0)
Supplement: Supplementary file 3 — Reporting Summary [file 41467_2020_18783_MOESM3_ESM.pdf]

nature research

Corresponding author(s): Hamish Gibs, Yang Liu

Last updated by author(s): Aug 21, 2020

Reporting Summary

Nature Research wishes to improve the reproducibility of the work that we publish. This form provides structure for consistency and transparency in reporting. For further information on Nature Research policies, see our Editorial Policies and the Editorial Policy Checklist.

Statistics

For all statistical analyses, confirm that the following items are present in the figure legend, table legend, main text, or Methods section.

n/a	Confirmed	
☐	☒	The exact sample size (n) for each experimental group/condition, given as a discrete number and unit of measurement
☐	☒	A statement on whether measurements were taken from distinct samples or whether the same sample was measured repeatedly
☐	☒	The statistical test(s) used AND whether they are one- or two-sided<br>*Only common tests should be described solely by name; describe more complex techniques in the Methods section.*
☒	☐	A description of all covariates tested
☐	☒	A description of any assumptions or corrections, such as tests of normality and adjustment for multiple comparisons
☐	☒	A full description of the statistical parameters including central tendency (e.g. means) or other basic estimates (e.g. regression coefficient) AND variation (e.g. standard deviation) or associated estimates of uncertainty (e.g. confidence intervals)
☐	☒	For null hypothesis testing, the test statistic (e.g. F, t, r) with confidence intervals, effect sizes, degrees of freedom and P value noted<br>*Give P values as exact values whenever suitable.*
☒	☐	For Bayesian analysis, information on the choice of priors and Markov chain Monte Carlo settings
☒	☐	For hierarchical and complex designs, identification of the appropriate level for tests and full reporting of outcomes
☒	☐	Estimates of effect sizes (e.g. Cohen's d, Pearson's r), indicating how they were calculated

Our web collection on statistics for biologists contains articles on many of the points above.

Software and code

Policy information about availability of computer code

Data collection	leidenalg 0.8.0, DXY-COVID-19-Crawler 0.0.0, Python 3.7.6, R 3.6.3
Data analysis	Custom code used for this analysis is publicly available in a Github repository, https://github.com/yangclaraliu/pandemic_travel_china.

For manuscripts utilizing custom algorithms or software that are central to the research but not yet described in published literature, software must be made available to editors and reviewers. We strongly encourage code deposition in a community repository (e.g. GitHub). See the Nature Research guidelines for submitting code & software for further information.

Data

Policy information about availability of data

All manuscripts must include a data availability statement. This statement should provide the following information, where applicable:

- Accession codes, unique identifiers, or web links for publicly available datasets
- A list of figures that have associated raw data
- A description of any restrictions on data availability

All data used in this study are publicly available. Analyses were conducted using a symmetrical matrix of movement flux data collected from the Qianxi Baidu 2020 movement map: https://qianxi.baidu.com/. The processed movement dataset is included in a publicly accessible repository: https://github.com/yangclaraliu/pandemic_travel_china. This research also relies on Covid-19 case count data and prefecture level hospital resource data from China, C. D. C. Public Health Science Data Center, http://www.phsciencedata.cn/Share/en/index.jsp, as well as prefecture level population data from China Statistical Yearbook 2018 http://www.stats.gov.cn/tjsj/ndsj/2018/indexeh.htm. Covid-19 case count data, prefecture level hospital resource data, and prefecture level population data require applications for use.

Field-specific reporting

Please select the one below that is the best fit for your research. If you are not sure, read the appropriate sections before making your selection.

☐ Life sciences ☒ Behavioural & social sciences ☐ Ecological, evolutionary & environmental sciences

For a reference copy of the document with all sections, see nature.com/documents/nr-reporting-summary-flat.pdf

Behavioural & social sciences study design

All studies must disclose on these points even when the disclosure is negative.

Study description	This study relies on quantitative human mobility data, healthcare resource data, population data, and Covid-19 case counts.
Research sample	Human mobility data is collected by Baidu Huiyan, a Location Bases Services provider. The dataset is openly available at : https://qianxi.baidu.com/. Covid-19 cases count data and prefecture level hospital resource data comes from China C. D. C Public Health Science Data Center. Prefecture level population data comes from China Statistical Yearbook 2018.
Sampling strategy	Analyses were conducted on aggregated mobility data from all users of Baidu Huiyan location services and referenced to individual Chinese prefectures. The sample size for this study is the population of Baidu Huiyan users actively reporting their location. For privacy reasons, this data is further processed by Baidu Huiyan into a "migration index" based on the volume of all travel in the network on a given day.
Data collection	Data was collected from the Baidu Qianxi web mapping interface https://qianxi.baidu.com/ by Hamish Gibbs and Yang Liu. Data for all Chinese prefectures were downloaded systematically, and the final dataset used in this analysis includes all movement flows available from the Baidu Qianxi web mapping interface.
Timing	Mobility data was collected from January 1st to March 1st.
Data exclusions	No data was excluded from this study.
Non-participation	This study relies on datasets aggregated by a location based services provider and therefore does not involve active observation of research participants.
Randomization	This study relies on datasets aggregated by a location based services provider using the population of all users actively sharing location data. The processing of the raw movement data does not permit randomization of specific populations of research subjects.

Reporting for specific materials, systems and methods

We require information from authors about some types of materials, experimental systems and methods used in many studies. Here, indicate whether each material, system or method listed is relevant to your study. If you are not sure if a list item applies to your research, read the appropriate section before selecting a response.

Materials & experimental systems

n/a	Involved in the study
☒	☐ Antibodies
☒	☐ Eukaryotic cell lines
☒	☐ Palaeontology and archaeology
☒	☐ Animals and other organisms
☒	☐ Human research participants
☒	☐ Clinical data
☒	☐ Dual use research of concern

Methods

n/a	Involved in the study
☒	☐ ChIP-seq
☒	☐ Flow cytometry
☒	☐ MRI-based neuroimaging
